# Preferred Attributes of Care Pathways for Obstructive Sleep Apnoea from the Perspective of Diagnosed Patients and High-Risk Individuals: A Discrete Choice Experiment

**DOI:** 10.1007/s40258-022-00716-1

**Published:** 2022-02-10

**Authors:** Andrea N. Natsky, Andrew Vakulin, Ching Li Chai-Coetzer, R. Doug McEvoy, Robert J. Adams, Billingsley Kaambwa

**Affiliations:** 1 Department of Health Economics, College of Medicine and Public Health, Flinders University, Health Sciences Building, Sturt Road, Bedford Park, Adelaide, SA 5042 Australia; 2 Adelaide Institute for Sleep Health/FHMRI Sleep Health, College of Medicine and Public Health, Flinders University, Adelaide, SA Australia; 3 National Centre for Sleep Health Services Research: A NHMRC Centre of Research Excellence, Flinders University, Adelaide, SA Australia; 4 Sleep and Circadian Research Group, Woolcock Institute of Medical Research, University of Sydney, Sydney, NSW Australia; 5 Respiratory and Sleep Services, Southern Adelaide Local Health Network, SA Health, Adelaide, SA Australia

## Abstract

**Background:**

The current healthcare system is challenged with a large and rising demand for obstructive sleep apnoea (OSA) services. A paradigm shift in OSA management is required to incorporate the preferences of diagnosed patients and individuals at high risk of OSA.

**Objectives:**

This study aimed to provide empirical evidence of the values and preferences of individuals diagnosed with OSA and high-risk populations regarding distinct OSA care pathway features.

**Methods:**

A discrete choice experiment was undertaken in two groups: those with a formal diagnosis of OSA (*n* = 421) and those undiagnosed but at high risk of having OSA (*n* = 1033). Participants were recruited from a large cross-sectional survey in Australia. The discrete choice experiment approach used mixed-logit regression models to determine preferences relating to eight salient features of the OSA management pathway, i.e. initial assessment provider, sleep study setting, diagnosis costs, waiting times, results interpretation, treatment options, provider of ongoing care and frequency of follow-up visits.

**Results:**

The findings indicate that all eight attributes investigated were statistically significant factors for respondents. Generally, both groups preferred low diagnostic costs, fewer follow-up visits, minimum waiting time for sleep study results and sleep specialists to recommend treatment. Management of OSA in primary care was acceptable to both groups and was the most preferred option by the high-risk group for the initial assessment, sleep study testing and ongoing care provision.

**Conclusions:**

The discrete choice experiment results offer a promising approach for systematic incorporation of patient and high-risk group preferences into the future design and delivery of care pathways for OSA management.

**Supplementary Information:**

The online version contains supplementary material available at 10.1007/s40258-022-00716-1.

## Key Points for Decision Makers

**Table:** 

Both diagnosed and undiagnosed, high-risk obstructive sleep apnoea groups preferred low diagnostic costs, fewer follow-up visits, a short waiting time for sleep study results and a sleep specialist to recommend treatment.
The primary care setting appears to be preferable and accessible for both groups, particularly those in the high-risk cohort.
The findings are useful for the future design and delivery of obstructive sleep apnoea management pathways that align with the values and preferences of patients and high-risk individuals in many settings.

## Introduction

Obstructive sleep apnoea (OSA) is a common sleep breathing disorder affecting 9–38% [1] of the general adult population and is associated with substantial health and economic costs to the individual and community [2, 3]. Improving access and efficiency of screening, diagnosis and therapy is crucial to managing patients and those at risk of developing OSA. However, the current system for managing OSA is complex and dependent on specialist services, unable to effectively meet the high and rising demand for diagnostic tests, with most individuals remaining undiagnosed and untreated in the community [4, 5].

In most settings, individuals are traditionally referred by a general practitioner (GP) to undergo a supervised sleep study in a sleep laboratory to establish a diagnosis of OSA before being managed by a sleep specialist. Despite the recent inclination towards ambulatory sleep testing, laboratory polysomnography remains the gold standard for diagnosis. However, polysomnography is costly, arduous and time consuming, creating access barriers compared with ambulatory limited-channel studies [6]. Following a sleep study, sleep clinicians usually prescribe treatments depending on OSA severity, symptoms and other clinical features including continuous positive airway pressure (CPAP), oral appliances and surgery [7–9]. Although CPAP is the most commonly recommended therapy, users often report physiological, medical and equipment-related issues, frequently resulting in poor acceptance and adherence [10, 11]. In contrast, patients may choose commercial outlets or pharmacies that offer portable OSA testing for the potential sale of CPAP and other treatment devices to expedite diagnosis and treatment [12, 13]. This direct interaction between private retailers and patients is contentious as it indeed reduces waiting time, yet can lead to a higher risk of improper treatment or discontinuation of therapy because of a lack of ongoing clinical care and patient education [14].

A paradigm shift in OSA management is required to increase the accessibility of medical sleep services whilst factoring in preferences and suitability for the end users. Understanding the preferences of potential and existing patients can help optimise the development of innovative OSA management pathways [15–17]. Previous studies have examined the preferences of patients, yet these studies only focused on OSA therapies [18–21] and management [22]. Less is known regarding different attributes that construct the comprehensive OSA care pathways, comprising options in healthcare providers for initial assessment, sleep study setting and costs, treatment initiation and options, waiting times, ongoing care frequency and provider. Using a discrete-choice experiment (DCE), this study was therefore designed to provide empirical evidence of the preferences of individuals diagnosed and suspected of OSA, regarding their prioritisation of the distinct OSA care pathways at the stages of diagnosis, treatment and ongoing care.

## Methods

A DCE was conducted with the objective of determining the relative importance between the salient attributes that make up an OSA care pathway. The DCE methodology is described in detail elsewhere [23, 24] and in the Appendix in the Electronic Supplementary Material (ESM).

### Survey Development and Administration

The development of attributes was guided through focus group discussions with the National Centre of Sleep Health Services Research-Centre of Research Excellence team within the Adelaide Institute for Sleep Health. Eight distinct attributes, each having five attribute levels were identified. The number of attribute levels ensures adequate perceptual differences and reflects realistic values to prevent potential bias due to the omission of relevant alternatives. The variable name used in the model for each attribute is shown in Table S1 of the ESM. Four blocks of seven choice sets (each with two alternatives, including an opt-out option) were developed (see Table S2 of the ESM, for an example choice set).

The DCE questionnaire was integrated into a cross-sectional web-based survey that sought to gather information on the health system experiences and preferences of individuals who were diagnosed or at high risk of OSA. The survey gathered information on demographics, OSA diagnosis status and symptoms, patient satisfaction, treatment motivations, options and compliance, and general health and quality of life (QoL) measured using EuroQol EQ-5D-5L [25] and the Functional Outcomes of Sleep Questionnaire-10 (FOSQ-10) [26]. Participants were adults ≥ 18 years of age recruited from an online panel by Dynata, a survey and market research company (Melbourne, VIC, Australia) in October 2019. The survey was approved by the Human Research Ethics Committee at Flinders University (Project 8435).

### Statistical and Econometric Approaches

Descriptive statistics and information regarding QoL were generated from the survey data. The QoL values were scored using respective algorithms from relevant instrument developers [27, 28]. The internal validity was approximated using a non-satiation test (see Table S3 of the ESM) [29, 30].

The hypothetical DCE choice sets were analysed according to the random utility theory, which assumed respondents would choose scenarios that maximised their utility. This utility (*U*), of alternative *j* for individual *n* in choice set *k,* is distinguished into two components where *X* is a vector of observed attributes, *β* is the vector of individual-specific parameters, and ${\varepsilon }_{njk}$ is an error term that is assumed to be independent and identically distributed as a type I extreme value, as shown in the following equation [31, 32]:

$$U_{njk} = \beta_{n} X_{jk} + \varepsilon_{njk}$$

The traditional conditional logit (clogit) was used initially to explore the preferences of the entire sample [33]. Other models were applied to circumvent the limitations of the clogit model, including (1) the heteroscedastic conditional logit (clogit-het), which allows testing for unequal variance (scale heterogeneity) [33]; (2) the uncorrelated and correlated mixed logit (MXL) that accounts for the unobserved taste or preference heterogeneity [34]; and (3) the generalised multinomial logit (G-MNL), which arguably controls for both unobserved random preference and scale heterogeneity [35–37]. The least preferred variable in the pooled sample was used as the reference point to generate a preference score ranking [37, 38]. As interaction terms were not incorporated in the DCE design, we used ‘main effects only’ to specify the models. ‘Cost’ and ‘follow-up frequency’ attributes were tested by the statistical significance of their quadratic term, with insignificant values suggesting the suitability of the linear assumptions [39].

In the traditional clogit model, each $\beta$ coefficient was estimated as a fixed parameter, assuming no unobserved preference heterogeneity. In the MXL model, all coefficients were assumed to be random and normally distributed, except for the cost attribute that assumed log normal distribution. The significance of the estimated standard deviation and likelihood ratio tests were examined to establish the overall contribution and suitability of the random parameters [40]. The Akaike information criterion and Bayesian information criterion, commonly used for model selection, were utilised to compare the statistical fit of all regression models whilst considering the existing heterogeneity in the data [41, 42].

The Swait–Louviere log-likelihood ratio test of equal parameters was undertaken to test for poolability across groups based on OSA diagnostic status [43]. Five other subgroups were considered, including sex, age, residential area, education and income level. Because of space constraints, the results for the subgroup analyses will be reported in a subsequent article. If the null hypothesis is rejected, then the subgroup models have significant statistical variation compared with the pooled sample and therefore need to be analysed separately [43]. The relative importance of the attributes of OSA care pathways was elicited by assessing the influence of attributes on individuals’ choices [44]. The projected probabilities of each mix of attribute levels being the preferred alternative were simulated using ranked estimations from the model coefficients [37, 38]. A significance level threshold of 5% was assumed, and all analyses were conducted in Stata 16 (StataCorp LLC, College Station, TX, USA) [45].

## Results

### Demographics and QoL Characteristics

Data relating to demographic characteristics and quality of life are displayed in Table 1. A total of 31,794 observations from 1516 people with no missing values were included in the analysis. Respondents who failed the non-satiation test (*n* = 60, 4%) were excluded from the analysis. The sample consisted of two groups of participants. The first group were individuals with a clinical diagnosis of OSA (*n* = 421) and the second group (*n* = 1033) had no formal diagnosis, but were categorised as at high risk for OSA based on a score of > 5 out of a maximum of 10 in the OSA-50 screening questionnaire [46]. These two groups of participants allowed exploration of the health service preferences of individuals who have previously engaged with an OSA care pathway and those with no prior experience.

**Table 1:** Demographic and quality-of-life details

Category	Diagnosed with OSA	Undiagnosed high-risk	Entire sample
	*n* = 421	*n* = 1033	*n* = 1454
Age groups in years, *n* (%)			
18–24	13 (3)	8 (1)	21 (1)
25–34	47 (11)	36 (3)	83 (6)
35–44	62 (15)	115 (11)	177 (12)
45–54	92 (22)	252 (24)	344 (24)
55–64	92 (22)	303 (29)	395 (27)
65–74	108 (26)	295 (29)	403 (28)
75 +	7 (2)	24 (2)	31 (2)
Sex, *n* (%)			
Male	234 (56)	458 (44)	692 (48)
Female	187 (44)	575 (56)	762 (52)
Education, *n* %			
Year 8 or below	8 (2)	14 (1)	22 (2)
Year 9	16 (4)	26 (3)	42 (3)
Year 10	41 (10)	116 (11)	157 (11)
Year 11	15 (4)	49 (5)	64 (4)
Year 12	52 (12)	158 (15)	210 (14)
Certificate	83 (20)	237 (23)	320 (22)
Diploma	63 (15)	141 (15)	214 (15)
Undergraduate and above	142 (34)	280 (27)	422 (29)
Prefer not to answer	1 (0)	2 (0)	3 (0)
Location, *n* (%)			
Metro	284 (67)	653 (63)	937 (64)
Regional	137 (33)	380 (37)	517 (36)
Quality of life, mean (SD)			
EQ5D-5L	0.65 (0.26)	0.74 (0.22)	0.71 (0.24)
EQ-VAS	63.06 (22.13)	66.57 (21.37)	65.55 (21.64)
FOSQ-10	16.74 (3.18)	17.80 (2.67)	17.49 (2.87)

*FOSQ-10* Functional Outcomes of Sleep Questionnaire-10, *OSA* obstructive sleep apnoea, *SD* standard deviation

Nearly half of the study sample was male (*n* = 732, 48%) and less than a third of the sample had been formally diagnosed with OSA (*n* = 421, 29%). The majority of the sample comprised people aged 45–74 years (*n* = 1142, 80%), educated beyond high school (*n* = 956, 66%) and who lived in a metropolitan area (*n* = 937, 64%). The mean score for EQ5D-5L for the entire sample was 0.71 (95% confidence interval [CI] 0.70–0.73) out of a maximum score of 1, indicating perfect health. A relatively higher score was observed in the undiagnosed high-risk OSA group (0.74 [95% CI 0.72–0.75]) and a lower score in the diagnosed OSA group (0.65 [95% CI 0.63–0.68]). A similar trend was found using the EQ-VAS (65.55 [95% CI 64.44–66.67]) for the entire sample (63.06 [95% CI 60.95–65.18] for confirmed OSA and 66.57 [95% CI 65.26–67.87] for high-risk OSA) and using the FOSQ-10 (17.49 [95% CI 17.35–17.64] for the whole sample, 16.74 [95% CI 16.44–17.05] and 17.80 [95% CI 17.64–17.96] for diagnosed and high-risk OSA groups, respectively).

### DCE Results

#### Comparisons between Regression Models and Subgroups

Four main models were estimated from the data: clogit, clogit-het, G-MNL and MXL. The statistical fit based on the entire sample, measured by Akaike information criterion and Bayesian information criterion scores, for the uncorrelated MXL model with 8000 Scrambled Halton draws was superior to the clogit and clogit-het, while the G-MNL and correlated MXL models were unable to be estimated because of convergence issues. The results of the clogit and clogit-het are available in Table S4 of the ESM. The results of the uncorrelated MXL models with 500 scrambled Halton draws using a full sample (*n* = 1514) and excluding respondents who failed non-satiation tests (*n* = 1454) can be found in Tables S6 and S7 of the ESM, respectively. The *X*^2^ statistics from the Swait–Louviere tests for equality of model parameters for the two groups (diagnosed and undiagnosed, high-risk OSA) were higher than the *X*^2^ critical value of 40.11 using a 5% level of significance and 27 degrees of freedom. Therefore, the Swait–Louviere tests indicate that analysing the data relating to the two subgroups as a pooled sample was inappropriate as higher log-likelihood for subgroup models relative to the *X*^2^ critical value suggested significant differences between preferences between both cohorts.

#### Important Attributes in Choice of OSA Management Pathway

The results of preferences for an OSA management pathway for diagnosed and undiagnosed, high-risk OSA groups are presented in Table 2. Wide variances in preferences were found in most attribute levels, signified by the significant standard deviation. The least preferred attributes used as references were: ‘dentist’ for initial assessment provider; ‘no sleep study/nowhere’ for test setting; ‘longer than 3 months’ for waiting time; ‘dentist or ear, nose, throat (ENT) surgeon’ for result interpretation; ‘throat surgery’ for treatment option; and ‘no one’ for the ongoing care provider.

**Table 2:** Uncorrelated mixed-logit regression estimates

Attribute and levels	Undiagnosed high-risk OSA	Diagnosed with OSA	Total sample
OSA initial assessment provider [ref: dentist]			
Self-assessed	0.475 (0.111)**	0.494 (0.128)**	0.460 (0.085)**
GP	1.433 (0.147)**	0.740 (0.162)**	1.168 (0.111)**
Pharmacy or CPAP shop	0.549 (0.139)**	0.353 (0.173)*	0.461 (0.108)**
No one	1.287 (0.436)**	− 0.631 (0.577)	0.645 (0.348)
Sleep study setting [ref: no sleep study]			
Private hospital	1.503 (0.416)**	1.465 (0.552)**	1.453 (0.333)**
GP	1.668 (0.346)**	1.188 (0.450)**	1.463 (0.276)**
Public hospital	1.268 (0.350)**	1.476 (0.466)**	1.336 (0.281)**
Pharmacy or CPAP shop	1.336 (0.381)**	1.364 (0.503)**	1.321 (0.305)**
Diagnostic cost			
(Continuous)	− 4.504 (0.109)**	− 5.014 (0.207)**	− 4.681 (0.101)**
Wait time [ref: longer than 3 months]			
No wait (same day)	0.943 (0.137)**	0.585 (0.166)**	0.797 (0.106)**
Within 1 week	0.819 (0.141)**	0.387 (0.167)*	0.648 (0.108)**
1 week to 1 month	0.476 (0.187)*	0.441 (0.213)*	0.474 (0.139)**
1 month to 3 months	0.241 (0.160)	0.047 (0.187)	0.160 (0.123)
Result interpretation and treatment recommendation provider [ref: dentist or ENT surgeon]			
Sleep physician	0.939 (0.162)**	0.858 (0.202)**	0.898 (0.126)**
GP	0.558 (0.147)**	0.369 (0.179)*	0.495 (0.114)**
Pharmacist or CPAP shop rep	0.424 (0.137)**	0.598 (0.170)**	0.481 (0.107)**
No one	0.407 (0.172)*	0.478 (0.208)*	0.416 (0.133)**
Treatment option [ref: throat surgery]			
CPAP therapy	0.646 (0.151)**	0.900 (0.194)**	0.747 (0.118)**
Mouthguard	0.853 (0.163)**	0.684 (0.195)**	0.782 (0.126)**
Lifestyle changes	0.854 (0.157)**	0.744 (0.189)**	0.811 (0.122)**
No treatment	0.454 (0.136)**	0.302 (0.167)	0.387 (0.106)**
OSA follow-up frequency			
(Continuous)	− 0.106 (0.038)**	− 0.069 (0.049)	− 0.082 (0.030)**
OSA ongoing care provider [ref: no one]			
Sleep physician	1.132 (0.218)**	1.098 (0.275)**	1.110 (0.170)**
GP	1.179 (0.250)**	0.775 (0.318)*	1.010 (0.196)**
Pharmacist or CPAP shop rep	0.795 (0.226)**	0.511 (0.291)	0.678 (0.179)**
Dentist or ENT surgeon	0.492 (0.248)*	0.532 (0.318)	0.478 (0.195)*
Standard deviations			
Diagnostic cost	1.247 (0.100)**	1.279 (0.173)**	1.292 (0.103)**
OSA follow-up frequency	0.229 (0.066)**	0.028 (0.159)	0.173 (0.060)**
OSA initial assessment provider			
Self-assessed	0.735 (0.174)**	0.317(0.309)	0.629 (0.146)**
GP	0.644 (0.237)**	− 0.109 (0.552)	0.536 (0.191)**
Pharmacist or CPAP shop rep	0.796 (0.198)**	0.864 (0.231)**	0.840 (0.151)**
No one	4.942 (0.289)**	3.855 (0.330)**	4.700 (0.222)**
Sleep study setting			
Private hospital	0.194 (0.494)	0.432(0.359)	0.251(0.386)
GP	0.450 (0.286)	0.326 (0.405)	0.523 (0.188)**
Public hospital	0.819 (0.164)**	0.571 (0.221)**	0.735 (0.134)**
Pharmacy or CPAP shop	0.058 (0.359)	− 0.173 (0.488)	0.079 (0.381)
Wait time			
No wait (same day)	− 0.004 (0.254)	0.071(0.468)	− 0.029 (0.205)
Within 1 week	0.230 (0.345)	0.032 (0.238)	0.022 (0.221)
1 week to 1 month	− 1.145 (0.348)**	− 0.782 (0.520)	− 0.987 (0.291)**
1 month to 3 months	0.539 (0.434)	0.744 (0.335)*	0.711 (0.239)**
Result interpretation and treatment recommendation provider			
Sleep physician	− 0.014 (0.204)	− 0.027 (0.393)	0.015 (0.174)
GP	0.320 (0.378)	− 0.011(0.606)	0.257 (0.325)
Pharmacist or CPAP shop rep	0.453 (0.315)	− 0.040 (0.474)	− 0.205 (0.426)
No one	1.261 (0.202)**	0.528 (0.366)	1.030 (0.157)**
Treatment option			
CPAP therapy	0.863 (0.235)**	-0.389 (0.380)	0.761 (0.184)**
Mouthguard	0.797 (0.276)**	0.851(0.277) **	0.836 (0.199)**
Lifestyle changes	0.728 (0.188)**	0.004 (1.314)	0.585 (0.163)**
No treatment	0.434 (0.293)	0.590 (0.239)*	0.587 (0.160)**
OSA ongoing care provider			
Sleep physician	0.598 (0.232)*	− 0.058 (0.420)	− 0.422 (0.200)*
GP	− 0.492 (0.276)**	− 0.041 (0.456)	− 0.400 (0.245)
Pharmacist or CPAP shop rep	0.460 (0.297)	0.788 (0.250)**	0.594 (0.197)**
Dentist or ENT surgeon	0.725 (0.201)**	0.554 (0.257)*	0.686 (0.154)**
LL	− 5235.21	− 2439.11	− 7751.18
AIC	10574.43	4982.217	15606.37
BIC	10831.32	5192.434	15881.03
*n*	1033	421	1454
Obs	21,693	8841	30,534
LL for S-L test^a^			153.722

In the simulation-based technique, 8000 Halton draws were run

*AIC* Akaike Information Criterion, *BIC* Bayesian Information Criterion, *CPAP* continuous positive airway pressure, *ENT* ear, nose and throat, *GP* general practice, *LL* Log-likelihood, *OSA* obstructive sleep apnoea, *ref* reference, *rep* representative, *SD* standard deviation

Figures are coefficients (standard errors), */**coefficient statistically significant at a 5%/1% level of significance

^a^S-L test = Swait-Louviere test is calculated using the sum of LL statistics of OSA diagnosed and high-risk groups subtracted from the LL of the pooled sample (− 7751.18). The *X*^2^ statistics from the S-L likelihood ratio tests for equality of model parameters for both groups (153.722) were higher than the *X*^2^ critical value of 40.113 (based on a 5% level of significance and 27 degrees of freedom). Hence, the data relating to both groups were analysed separately as analysing by the pooled sample is inappropriate

In the sample of individuals with diagnosed OSA, participants expressed a preference for a GP as the person for the initial OSA assessment. They also preferred to take the sleep study in a public hospital setting with no wait (same day) for the test and at a minimum out-of-pocket cost. Additionally, a sleep specialist was preferred to be the person who interprets the sleep study results and provides ongoing care, with CPAP as the most favoured treatment option. The ongoing care frequency was not statistically significant in this group.

In contrast, all of the eight attributes were significant in the undiagnosed high-risk OSA sample. This group also preferred a GP as the initial assessment provider, low diagnostic costs, no waiting time to have a sleep study, fewer ongoing care visits and a sleep specialist to interpret their sleep study results. However, they would rather have their sleep study and ongoing care provided in a primary care setting, and opted for lifestyle changes, followed closely by a mouthguard as the treatment of choice.

The predicted probabilities associated with selecting the five highest-ranked OSA management pathways for each subgroup in both OSA-diagnosed and high-risk groups are presented in Table S5 of the ESM. The overlapping 95% CI of the preference score and the probabilities indicate no statistically significant differences between the top five preferred OSA management pathways, suggesting that participants highly valued all of the high-ranked packages. In general, all of the attributes were deemed essential. However, the relative importance is different for the two groups, except for the diagnostic costs and ongoing care frequency, which were preferred to be low and the initial assessment and result interpretation providers who remain unvaried in both groups.

## Discussion

The findings from this study represent the first empirical evidence about the stated preferences for OSA management pathways taken from two groups: diagnosed with OSA and undiagnosed but at high risk of OSA. These results are highly pertinent for sleep health professionals and policymakers as they present valid evidence of valued preferences that can inform the future redesign of care pathways for OSA from the consumer perspective. The substantial unobserved heterogeneity signified by the significant standard deviation in most attribute levels (see Table 2) indicates how people can distinctively value each alternative of the OSA management pathway.

In most countries, the first point of contact for OSA assessment can be through a health provider such as the GP, dentist, or pharmacist or by self-assessment. Based on the DCE results, the OSA-diagnosed group favoured having their initial assessment conducted by a GP in a primary care setting. Similarly, the GP was also the highest ranked option for the high-risk OSA group, followed by ‘no-one’. Public awareness of OSA is believed to have gradually improved over the last few decades [47]. However, the fact that the high-risk groups chose ‘no-one’ as their second-best alternative to investigate their OSA rather than health professionals or self-assessment presumably reflects the lack of awareness related to negative outcomes from untreated OSA. Further evidence of the distinct preferences of people with diagnosed vs undiagnosed OSA is observed through their choices in waiting times. People diagnosed with OSA had a relatively higher tolerance than the high-risk sample who preferred the shortest waiting time. Again, this may reflect a prior experience that people in the diagnosed group have had in the delivery of sleep services and that they are conscious there is typically a long waiting period before a sleep study, to obtain results and commence therapy. The DCE finding is aligned with a recent study that found the current state of awareness and knowledge of OSA among the general community is low [48].

Second to the GP, pharmacists and CPAP shop representatives were also positively preferred by both groups as the initial assessment provider. A study in the USA showed that pharmacists often use screening processes to identify patients at high risk of OSA and provide advice to those requiring further investigation [49]. Despite the possibility of better access to sleep study testing through a pharmacy or CPAP retailer, preliminary assessment at these locations often bypasses medical professional involvement and patients may be left with minimal advice and commitment beyond diagnosis and CPAP purchase [14]. However, dentists were the least preferred, particularly for those with undiagnosed high-risk OSA. A recent literature review regarding the role of dentists in the care of patients with OSA revealed that dentists can manage OSA to a certain degree, yet they lack clinical experience and rarely referred patients to sleep physicians for further treatment [50, 51].

Stark differences were found in the sleep study setting preference between the two cohorts. The strong inclination towards hospital-based services in the diagnosed group could reflect their retrospective experience of being referred to a hospital for a sleep study and not necessarily what they would have preferred, as conversely, the high-risk group preferred a GP as their first choice. Unfortunately, sleep studies are not widely available in primary care settings in most countries [17, 52–55]. The positive tendency towards the primary care setting could be because GPs are readily available and accessible in the community, also demonstrated by the strong preference towards a GP as an initial assessment provider. Furthermore, both groups preferred low diagnostic costs. This presents an opportunity to investigate the cost effectiveness of limited channel studies, a more accessible and affordable alternative OSA diagnostic test, to expand the availability and funding for sleep studies in general practice [52–55]. Provision of home ambulatory testing by primary care could potentially improve access and convenience for individuals suspected of OSA and shorten waiting times associated with hospital services [56, 57].

Following a sleep study, both samples were faced with a scenario where they had to choose a health professional that they most preferred to interpret their diagnostic results and recommend treatment. Although a GP was very popular for the initial assessment and sleep study setting attributes, the preference wanes compared with a sleep specialist who was a dominant choice in both groups. Similarly, the sleep specialist was also the most favoured health professional to provide ongoing care in both groups, who also preferred fewer follow-ups. However, the number of people affected by OSA is becoming more prevalent, creating an immense unmet demand for sleep specialist services [1, 5]. A continued focus on management pathways that utilise specialist care can only create further access restrictions and escalate waiting times for disease management [14, 58].

Transitioning the focus from specialist services to primary care seems appropriate as there is the potential capacity to rectify the undersupply issue of sleep physicians. Existing evidence suggests that OSA management in the primary care setting does not result in worse health outcomes and compliance than hospital-based care [52, 53, 59]. The DCE findings also highlighted a significant preference for GPs as initial care providers in both cohorts. Despite not being the first choice for interpreting results and ongoing care management, the preference for a GP was still robustly positive and popular in both cohorts. However, numerous challenges in managing sleep disorders in primary care remain. General practitioners currently have limited expertise, and lack the necessary management tools and clear clinical pathways to refer complex or treatment-resistant patients [52]. There are still some instances where primary care physicians fail to recognise, make reliable diagnoses and refer high-risk individuals for further management [60]. Calculated measures need to be taken to equip primary care providers with the required skills and knowledge to competently manage OSA [61].

Regarding treatment options, both samples provided positive responses toward all treatments, except for the surgical approach, which scored lower than no treatment in both samples. The negative association could imply a distaste for invasive procedures, related potential risks and high out-of-pocket costs. The least intrusive approaches, such as lifestyle changes and mouthguards, were favoured mainly by the high-risk group. On the contrary, despite potential issues related to usage [10, 11], CPAP remains the most highly preferred choice in the OSA diagnosed group, followed by lifestyle changes and mouthguards. This preference could reflect past experiences of the OSA diagnosed group as CPAP is the most commonly prescribed therapy [6]. The marked preference for CPAP could also suggest the valued importance of treatment efficacy compared to convenience compared with those in the undiagnosed high-risk sample, who primarily preferred a mouthguard.

### Limitations of the Study

This study utilised a robust methodology and included a more comprehensive range of attributes than previous research studies, capturing the preferences for an OSA care pathway in a large representative sample with no missing data [20–22]. However, some limitations remain. First, the study predominantly used results from the uncorrelated MXL version, disallowing random scale heteroscedasticity, which could arguably be a source of bias in the estimate [62]. Nevertheless, failure in accounting for heterogeneity in preference as captured by the uncorrelated MXL is of greater empirical consequence relative to the inability to account for unequal variances and scale heterogeneity in terms of behavioural outputs [36]. Furthermore, recent research suggests that the G-MNL model is a constrained form of MXL, which claims to only allow for scale heterogeneity, yet captures other sources of correlation in the scale parameter [63]. Second, the survey was administered online, which could cause inaccuracy relative to face-to-face interviews. The non-satiation test was utilised to exclude ineligible respondents from the analysis, and the overall sample was able to produce statistically significant results. Finally, the DCE approach limits the number of attributes that can be included in its design. Further investigation into stated and revealed preference studies that incorporate nursing and telehealth services are warranted to provide insights into the potential of such mechanisms in OSA management.

## Conclusions

Marked by access barriers to diagnostic sleep services, treatment delays, and low treatment acceptance and adherence, current OSA care pathways remain complex and challenging to navigate for many patients. Obtaining input about patient preferences is essential in developing new sleep service delivery models for OSA that can mitigate the enduring problems and improve continuity of care [18]. Furthermore, consideration of a patient’s health literacy is vital in ensuring that they are making informed choices and remaining active participants in managing their chronic condition [14]. Generally, both diagnosed and undiagnosed high-risk OSA groups preferred low diagnostic costs, fewer follow-up visits, a short waiting time for sleep study results and a sleep specialist to recommend treatment. Given the substantial unmet demand for OSA services due, in part, to the scarcity of sleep specialists, sharing the burden with primary care and utilising community-based management models can broaden access to sleep study testing and expedite OSA diagnosis and therapy. Although further steps are needed to improve education and training, the primary care setting was acceptable to both cohorts and was the most preferred option by the high-risk undiagnosed group for the initial assessment provider, sleep study testing and provision of ongoing care. Overall, the result of the DCE offers a promising approach for systematic incorporation into the future design and delivery of care pathway for OSA.

## Supplementary Information

Below is the link to the electronic supplementary material.

Supplementary file1 (DOCX 73 kb)

## Declarations

### Availability of Data and Material

The datasets generated and analysed during the current study are not publicly available as they may be disclosive, which would contravene ethical approval. Appropriately anonymised data and relevant code are available from the corresponding author on reasonable request.
