# Supplementary material for: Preferred Attributes of Care Pathways for Obstructive Sleep Apnoea from the Perspective of Diagnosed Patients and High-Risk Individuals: A Discrete Choice Experiment
Source: Appl Health Econ Health Policy. 2022 Feb 10;20(4):597–607. doi: 10.1007/s40258-022-00716-1 (PMC9206920; doi:10.1007/s40258-022-00716-1)
Supplement: Supplementary file 1 — Supplementary file1 (DOCX 73 kb) [file 40258_2022_716_MOESM1_ESM.docx]

## Applied Health Economics and Health Policy

### Preferred Attributes of Care Pathways for Obstructive Sleep Apnoea from the Perspective of Diagnosed Patients and High-risk Individuals: A Discrete Choice Experiment

### **Supplementary Materials**

Andrea N Natsky, MPH1,3, Andrew Vakulin, PhD2,3,4, Ching Li Chai Coetzer, MBBS, FRACP, GCPH, PhD 2,3,5 , R. Doug McEvoy, B Med Sc, MBBS, FRACP, MD, PhD 2,3,5, Robert J. Adams, MBBS, MD, FRACP, FRCP, PhD 2,3,5, Billingsley Kaambwa, BA, MA, PhD 1,3

## Affiliations:

^1^ Department of Health Economics, College of Medicine and Public Health, Flinders University

^2^Adelaide Institute for Sleep Health / FHMRI Sleep Health, College of Medicine and Public Health, Flinders University

^3^National Centre for Sleep Health Services Research: A NHMRC Centre of Research Excellence, Flinders University, <https://www.ncshsr.com/>

^4^Sleep and Circadian Research Group, Woolcock Institute of Medical Research, University of Sydney

^5^Respiratory and Sleep Services, Southern Adelaide Local Health Network, SA Health

## Corresponding author:

Andrea N Natsky, Health Economics, Flinders University, College of Medicine and Public Health, Health Sciences Building, Sturt Road, Bedford Park, South Australia 5042

Phone: +618 7221 8456

Email: [andrea.natsky@flinders.edu.au](mailto:andrea.natsky@flinders.edu.au)

## Funding

This was not an industry-funded study. This study was supported through the Australian Government Research Training Program, and National Health and Medical Research Council: CRE National Centre of Sleep Health Services Research (Grant ID 1134954).

## Conflicts of interest

All authors report no conflicts of interest.

## Appendix

## Methods

### Development of attributes and DCE survey administration

The DCE attributes were entered into Ngene software ^1^ to design a D-efficient D-error measure (with zero priors), which reduce variation around the parameter estimates by minimising estimated standard errors and the determinant of the covariance matrix ^2^. The variable name used in the model for each attribute is shown in Supplementary Table S1. Four blocks of seven choice sets (each with two alternatives, including an opt-out option) were developed to minimise time and cognitive demand for respondents. Main effects design was utilised as commonly used by other DCE studies ^3-6^.

Survey invitations were sent through various platforms including email, telephone alerts, banner and online messaging. A three-stage randomisation process is used to match a potential participant with a survey they are likely to complete and minimise the risk of bias ^7^. To reduce respondent burden, the study participants were randomised into four groups, where each cohort was given one of the four versions of the DCE questionnaire. A wide selection of participants was purposefully drawn from Dynata’s panel to maximise variation of respondents’ characteristics. As we were specifically looking for participants diagnosed with OSA or at high-risk of having OSA, the invitation to participate was restricted to participants over 35 years of age as the risk of having OSA increases with age. The messages were varied, including invitations to provide an opinion, earn cash or prizes (value of this is approximately $1) or let your voice be heard. To avoid self-selection bias, specific project details were not included in the invitation. Instead, participants were invited to “take a survey”. The panel was sampled in order to generate a study sample that matches the Australian Bureau of Statistics estimates on sex, state and regional representation.

### Statistical and econometric approaches

In the traditional clogit model, each $\beta$ coefficient was estimated as a fixed parameter, suggesting a homogenous preference. The scale parameters derived from clogit-het can be converted using the following equation: $\sigma_{i}=exp({\theta z}_{i})$, where $z_{i}$ is a vector of observed individual characteristics and $\theta$ represents a parameter vector that captures the effect of individual characteristics on the error variance^8^. In contrast, the $\beta_{n}$ in the MXL models was specified as a random parameter (i.e. $\beta_{n}=\beta+ \eta_{n}$, where $\eta_{n}$ is a vector of individual n-specific deviations from the mean) ^9^. The mean and standard deviation for each random $\beta$ coefficient was estimated following a normal distribution, representing distinctive individual tastes ^9^.

In the MXL, all coefficients were assumed to be random and normally distributed, except for cost attribute that assumed log normal distribution. The significance of the estimated standard deviation and likelihood-ratio tests were examined to establish the overall contribution and suitability of the random parameters ^10^. The default simulation estimation used in the MXL model was Halton sequences which had a restriction of 20 random variables ^11^. As the data exceeds this limit, pseudorandom and scrambled Halton sequences were utilised to calculate the high dimension model parameters. Scrambled Halton draws were considered an improvement of the traditional pseudorandom and standard Halton sequence, as well as matching the best-performing scrambled Sobol draws in terms of models with high dimensionality integration ^12,13^. Correlation between parameters was accounted for in the MXL model and tested using the joint significance of diagonal elements of the covariance matrix for the correlated-attribute models using a likelihood-ratio test under the null of an unrelated coefficient model and goodness of fit tests ^11^. The Akaike information criterion (AIC) and Bayesian information criterion (BIC), commonly used for model selection, were utilised to compare the statistical fit of all regression models whilst considering the existing heterogeneity in the data ^14,15^. Five other subgroups were considered, including gender, age, residential area, education and income level. Results for the subgroup analyses will be reported separately.

## Results

### Demographics and QoL characteristics

From a total of 3,812 individuals sampled, completed surveys were returned by 1,516 individuals who qualified as having a diagnosis of OSA or being at high-risk for OSA (Estimated models from the full sample is provided in Table S6). Four versions of the DCE questionnaire were distributed evenly amongst the respondents. Sixty people (4%) failed the internal consistency test and were excluded from the regression modelling following analysis of statistical fit comparison of both inclusion and exclusion of irrational responses. A further two respondents with gender registered as ‘other’ were excluded from the analysis as the relatively low number prevented proper analysis to be done based on the subgroup.

The questionnaire was completed by a broad scope of the general adult population aged 18 to 75 years and over with a proper gender balance (male 48%, female 52%). The preponderance of the sample consisted of people aged 45 to 74 years old (1,142, 80%), which reflects the higher prevalence of OSA in older individuals^16^. The average QoL scores measured by EQ-5D-5L, EQ-VAS, and FOSQ-10 for the diagnosed group were relatively lower than those of the high-risk groups. The respondents in our total sample had noticeably lower QoL as measured by the EQ-5D-5L (total sample mean score of 0.71) and EQ-VAS (65.55) compared with the findings from a South Australian population norms with mean scores of 0.91 and 78.55 respectively ^17^. The lower scores found are anticipated as the entire sample comprises people who have had a diagnosis of OSA or are at high risk of OSA, therefore, associated with lower QoL^18,19^. To our knowledge, there has been no other representative mean scores available for the FOSQ-10, making it challenging to make a valid comparison between the values found in this study and those for the general population.

### DCE results

#### Comparisons between regression models and subgroups

Four main models were applied to the data: clogit, clogit-het, G-MNL and MXL. The statistical fit based on the entire sample, measured by AIC and BIC scores, for the uncorrelated MXL model was superior than the clogit and clogit-het, implying that the former was an improvement over the latter two. The G-MNL and correlated MXL models were unable to be fully estimated for the subgroups due to lack of convergence. In the preliminary analysis, using a low number of scrambled Halton draws (nrep = 5), the uncorrelated MXL still had the highest statistical fit relative to G-MNL and correlated MXL models in corresponding subgroups. Consequently, only DCE results from the best-fit uncorrelated MXL models are reported with 8000 scrambled Halton draws. The results of the clogit and clogit-het are available in Supplemental Table S4, while the results of the penultimate uncorrelated MXL models with 500 scrambled Halton draws can be found in Supplemental Table S7.

**Table S1** Attribute and attribute levels

| Attribute | Description | Attribute levels | Variable name used in models |
| --- | --- | --- | --- |
| OSA initial assessment provider | If you suspected that you had obstructive sleep apnea, who would you see for an initial assessment? | No one  Self-assessed  GP  Pharmacy or CPAP shop  Dentist | initialax_noone  initialax_sa  initialax_gp  initialax_pharma  initialax_dentist |
| Sleep study setting | Where would you most likely go for a sleep study? | Nowhere  Private hospital  GP  Public hospital  Pharmacy or CPAP shop | setting_nowhere  setting_private  setting_gp  setting_public  setting_pharma |
| Diagnostic test cost | How much would you be willing to pay out-of-pocket towards the cost of a sleep study to investigate sleep apnea? | AU$0  AU $50  AU $100  AU $150  AU $200 | dcost (continuous variable) |
| Waiting time for test results and treatment initiation | How long are you willing to wait for the diagnostic test results and start therapy? | No wait (same day)  Within 1 week  1 week to 1 month  1 month to 3 months  Greater than 3 months | waittime_nowait  waittime_1week  waittime_1month  waittime_3month  waittime_longer |
| Results interpretation and treatment recommendation | Who would you prefer to interpret your sleep study result and make your treatment recommendation? | No one  GP  Sleep specialist doctor  Pharmacy or CPAP shop  Dentist or ENT surgeon | resultint_noone  resultint_gp  resultint_sleepspec  resultint_pharma  resultint_dentistent |
| Treatment option | Which treatment would be your preferred option to manage your OSA? | No treatment  CPAP therapy (mask)  Mouthguard  Lifestyle changes  Throat surgery | tx_notreat  tx_cpap  tx_mouthguard  tx_lifestyle  tx_mouthguard |
| OSA ongoing care frequency | How often do you want to be followed-up after initiating treatment for your OSA in a single year? | 0 times/ year  1 time  2 times/ year  3 times/ year  4 times/ year | fufrq (continuous variable) |
| OSA ongoing care provider | Who should be primarily responsible for ongoing care of your OSA? | No one  GP  Pharmacy or CPAP shop  Sleep specialist doctor  Dentist or ENT surgeon | fupro_noone  fupro_gp  fupro_pharma  fupro_sleepspec  fupro_dentistent |

Note: dummy coding utilised for all variables except for Diagnostic test cost ‘dcost’ and OSA ongoing care frequency ‘fufrq’

*CPAP=* Continuous positive airway pressure; *ENT=* Ear, Nose and Throat; *GP=* General practice; *OSA*= Obstructive sleep apnoea

**Table S2** Excerpt of OSA sleep service pathway choice set

For each of the choices below, which package would you choose?

| Attribute | Sleep Service 1  (Alternative 1) | Sleep Service 2  (Alternative 2) | No Sleep Service  (opt-out) |
| --- | --- | --- | --- |
| 1. OSA initial assessment provider | Dentist | Self-assessed | No one |
| 1. Sleep study setting | Pharmacy or CPAP shop | Public hospital | Nowhere |
| 1. Diagnostic test cost | $150 | $0 | $0 |
| 1. Waiting time for test results and treatment initiation | 1 week to 1 month | 1 week to 1 month | No wait |
| 1. Results interpretation and treatment recommendation | Dentist or ENT | Sleep physician | No one |
| 1. Treatment option | No treatment | Mouthguard | No treatment |
| 1. OSA ongoing care frequency | 3 times/ year | 3 times/ year | 0 times/ year |
| 1. OSA ongoing care provider | Sleep physician | Sleep physician | No one |

Which Sleep Service would you choose? Sleep Service 1 Sleep Service 2 No Sleep Service

*(please tick one box only)* ⬜ ⬜ ⬜

**Table S3** Non satiation test^a^

### **A survey of patients’ views concerning the characteristics of sleep service models for managing obstructive sleep apnea (OSA)**

We would like to invite you to participate in the following research survey. We are asking about your preferences for different ways of providing sleep services for managing OSA. This section will present hypothetical scenarios. We ask you to compare and carefully consider each attribute of offered options. After that, please select which of the two packages you would most prefer if they were available.

The first one is a practice example of choices that you might encounter in your everyday life. In this case, you are deciding on going for your OSA management at two different sleep services. Please consider the characteristics of each sleep service and then select the one you would prefer most.

**Practice example**

| **Attribute** |  | **Sleep Service 1**  **(Alternative 1)** |  | **Sleep Service 2**  **(Alternative 2)** |  | **No Sleep Service**  **(opt-out)** |
| --- | --- | --- | --- | --- | --- | --- |
| 1. Distance of sleep clinic from your home |  | 100km away |  | 10km away |  | I do not want to attend a sleep clinic |
| 1. Waiting time to be seen at a sleep clinic |  | Long waiting time |  | No waiting time |  |  |
| 1. Cost of treatment |  | $200 |  | $50 |  | $0 |
| 1. Effectiveness of treatment |  | 50% effective |  | 100% effective |  | No treatment |

Which Sleep Service would you choose? Sleep Service 1 Sleep Service 2 No Sleep Service

*(please tick one box only)* ⬜ ⬜ ⬜

**Table S4** Conditional Logit regression results^a^

|  | | Main Effect Traditional Model (Clogit) | Clogit Heteroscedastic diagnostic status subgroup |
| --- | --- | --- | --- |
| Mean | | | |
| Diagnostic cost | | -0.006**  (0.000) | -0.001**  (0.000) |
| OSA follow up frequency | | -0.040*  (0.017) | -0.007*  (0.003) |
| OSA initial assessment provider | Self-assessed | 0.282**  (0.051) | 0.047**  (0.014) |
|  | GP | 0.631**  (0.058) | 0.111**  (0.028) |
|  | Pharmacist or CPAP shop rep | 0.306**  (0.059) | 0.054**  (0.016) |
|  | No one | 1.087**  (0.191) | 0.190**  (0.056) |
| Sleep study setting | Private Hospital | 0.803**  (0.192) | 0.122**  (0.045) |
|  | GP | 0.795**  (0.164) | 0.129**  (0.042) |
|  | Public Hospital | 0.735**  (0.168) | 0.108**  (0.040) |
|  | Pharmacy or CPAP shop | 0.652**  (0.182) | 0.098*  (0.040) |
| Wait time | No wait (same day) | 0.445**  (0.056) | 0.077**  (0.021) |
|  | Within 1 week | 0.371**  (0.058) | 0.065**  (0.018) |
|  | 1 week to 1 month | 0.308**  (0.077) | 0.052**  (0.018) |
|  | 1 month to 3 months | 0.196**  (0.065) | 0.035*  (0.014) |
| Result interpretation and treatment recommendation provider | Sleep physician | 0.365**  (0.066) | 0.061**  (0.019) |
|  | GP | 0.221**  (0.060) | 0.039**  (0.014) |
|  | Pharmacist or CPAP shop rep | 0.241**  (0.056) | 0.039**  (0.014) |
|  | No one | 0.184*  (0.076) | 0.027  (0.015) |
| Treatment option | CPAP therapy | 0.402** | 0.057** |
|  |  | (0.063) | (0.018) |
|  | Mouthguard | 0.437**  (0.067) | 0.070**  (0.021) |
|  |  |  |  |
|  | Lifestyle changes | 0.448**  (0.061) | 0.072**  (0.021) |
|  |  |  |  |
|  | No treatment | 0.196**  (0.060) | 0.032*  (0.013) |
|  |  |  |  |
| OSA ongoing care provider | Sleep physician | 0.629**  (0.102) | 0.102**  (0.031) |
|  | GP | 0.519**  (0.117) | 0.087**  (0.029) |
|  | Pharmacist or CPAP shop rep | 0.413**  (0.107) | 0.072**  (0.025) |
|  | Dentist or ENT surgeon | 0.226  (0.117) | 0.035  (0.022) |
| Heteroscedasticity | | | |
| OSA diagnostic status (yes/ no) | |  | 0.958** |
|  |  |  | (0.121) |
| LL^b^ | | -10566 | -10507.4 |
| AIC^c^ | | 21184.08 | 21068.77 |
| BIC^d^ | | 21321.42 | 21211.39 |
| N | | 1,454 | 1,454 |
| Observations | | 30,534 | 30534 |

*CPAP=* Continuous positive airway pressure; *ENT=* Ear, Nose and Throat; *GP=* General practice; *OSA*= Obstructive sleep apnoea; *SD*= standard deviation

Note: figures are coefficient (standard errors)

^a^ In the simulation-based technique, 500 Halton draws were run

^b^ LL = Log-likelihood

^c^ AIC = The Akaike information criterion

^d^ BIC = Bayesian information criterion

*/** Coefficient statistically significant at 5%/ 1% level of significance

**Table S5** Predicted probabilities for the top 5 preferred OSA management pathway

| Undiagnosed, high risk OSA | | | | | | | | | | |
| --- | --- | --- | --- | --- | --- | --- | --- | --- | --- | --- |
| Initial assessment provider | **Sleep study setting** | **Diagnostic test costs** | **Waiting time** | **Results interpretation** | **Treatment option** | **Ongoing care frequency** | **Ongoing care provider** | **Preference score (95% CI)^a^** | **Probability (95% CI)^b^** | **Rank** |
| GP | GP | Low | No wait | Sleep specialist | Lifestyle changes | Less | GP | 2.406 (1.046, 3.766) | 0.204 (0.189, 0.218) | 1 |
| GP | GP | Low | No wait | Sleep specialist | Mouthguard | Less | GP | 2.405 (1.015, 3.794) | 0.204 (0.186, 0.221) | 2 |
| GP | GP | Low | No wait | Sleep specialist | Lifestyle changes | Less | Sleep specialist | 2.359 (1.088, 3.630) | 0.200 (0.182, 0.218) | 3 |
| GP | GP | Low | No wait | Sleep specialist | Mouthguard | Less | Sleep specialist | 2.358 (1.058, 3.658) | 0.200 (0.180, 0.219) | 4 |
| GP | GP | Low | Within 1 week | Sleep specialist | Lifestyle changes | Less | GP | 2.282 (0.899, 3.665) | 0.193 (0.173, 0.213) | 5 |
| OSA diagnosed | | | | | | | | | | |
| Initial assessment provider | **Sleep study setting** | **Diagnostic test costs** | **Waiting time** | **Results interpretation** | **Treatment option** | **Ongoing care frequency** | **Ongoing care provider** | **Preference score (95% CI)^a^** | **Probability (95% CI)^b^** | **Rank** |
| GP | Public hospital | Low | No wait | Sleep specialist | CPAP | Less | Sleep specialist | 0.575 (-1.111, 2.262) | 0.237 (0.048, 0.426) | 1 |
| GP | Private hospital | Low | No wait | Sleep specialist | CPAP | Less | Sleep specialist | 0.564 (-1.373, 2.502) | 0.232 (0.114, 0.351) | 2 |
| GP | Pharmacy or CPAP shop | Low | No wait | Sleep specialist | CPAP | Less | Sleep specialist | 0.463 (-1.364, 2.291) | 0.191 (0.038, 0.343) | 3 |
| GP | Public hospital | Low | No wait | Sleep specialist | Lifestyle changes | Less | Sleep specialist | 0.419 (-1.202, 2.041) | 0.237 (0.048, 0.426) | 4 |
| GP | Private hospital | Low | No wait | Sleep specialist | Lifestyle changes | Less | Sleep specialist | 0.408 (-1.450, 2.266) | 0.237 (0.048, 0.426) | 5 |

*CPAP=* Continuous positive airway pressure; *GP=* General practice; *OSA*= Obstructive sleep apnoea;

^a^ all preference scores were computed by summing up the model coefficients for every combination of attribute levels. 95% CI = 95% confidence interval

^b^ the probability that each mixture of attribute levels package is the most preferred scenario was estimated by dividing the preference score for the certain preferred packaged by the sum of all five highest preference scores. 95% CI = 95% confidence interval.

**Table S6** Uncorrelated Mixed Logit regression estimates [all participants, n=1514] ^a^

| Attribute and levels | | | Undiagnosed, high-risk OSA | Diagnosed with OSA | | Total sample | |
| --- | --- | --- | --- | --- | --- | --- | --- |
| OSA initial assessment provider  [ref: dentist] | Self-assessed | | 0.519** (0.109) | 0.490** (0.123) | | 0.470** (0.082) | |
|  | GP | | 1.373** (0.135) | 0.645** (0.156) | | 1.065** (0.103) | |
|  | Pharmacy or CPAP shop | | 0.549** (0.136) | 0.332* (0.169) | | 0.458** (0.106) | |
|  | No one | | 1.514** (0.420) | -0.501 (0.537) | | 0.848** (0.325) | |
| Sleep study setting  [ref: no sleep study] | Private Hospital | | 1.354** (0.400) | 1.208* (0.526) | | 1.273** (0.318) | |
|  | GP | | 1.550** (0.330) | 1.042* (0.427) | | 1.316** (0.264) | |
|  | Public Hospital | | 1.200** (0.337) | 1.301** (0.444) | | 1.224** (0.270) | |
|  | Pharmacy or CPAP shop | | 1.284** (0.367) | 1.242** (0.481) | | 1.260** (0.292) | |
| Diagnostic cost | (continuous) | | -0.016** (0.001) | -0.010** (0.001) | | -0.014** (0.001) | |
| Wait time  [ref: longer than three months] | No wait (same day) | | 0.914** (0.132) | 0.457** (0.157) | | 0.738** (0.100) | |
|  | Within 1 week | | 0.761** (0.138) | 0.273 (0.165) | | 0.587** (0.105) | |
|  | 1 week to 1 month | | 0.426* (0.183) | 0.382 (0.204) | | 0.447** (0.133) | |
|  | 1 month to 3 month | | 0.227 (0.157) | -0.063 (0.188) | | 0.109 (0.119) | |
| Result interpretation and treatment recommendation provider  [ref: dentist or ENT surgeon] | Sleep physician | | 0.931** (0.157) | 0.766** (0.191) | | 0.890** (0.122) | |
|  | GP | | 0.567** (0.143) | 0.362* (0.173) | | 0.516** (0.110) | |
|  | Pharmacist or CPAP shop rep | | 0.470** (0.133) | 0.640** (0.167) | | 0.543** (0.103) | |
|  | No one | | 0.418* (0.167) | 0.489* (0.199) | | 0.425** (0.128) | |
| Treatment option  [ref: throat surgery] | CPAP therapy | | 0.694** (0.145) | 0.858** (0.183) | | 0.735** (0.111) | |
|  | Mouthguard | | 0.906** (0.156) | 0.711** (0.190) | | 0.792** (0.120) | |
|  | Lifestyle changes | | 0.854** (0.150) | 0.724** (0.184) | | 0.775** (0.114) | |
|  | No treatment | | 0.448** (0.132) | 0.313 (0.162) | | 0.363** (0.100) | |
| OSA follow up frequency | (continuous) | | -0.107** (0.037) | -0.047 (0.046) | | -0.080** (0.029) | |
| OSA ongoing care provider  [ref: no one] | Sleep physician | | 1.112** (0.209) | 0.982** (0.250) | | 1.081** (0.162) | |
|  | GP | | 1.086** (0.240) | 0.612* (0.297) | | 0.929** (0.188) | |
|  | Pharmacist or CPAP shop rep | | 0.785** (0.221) | 0.488 (0.269) | | 0.668** (0.172) | |
|  | Dentist or ENT surgeon | | 0.442 (0.243) | 0.427 (0.295) | | 0.468* (0.187) | |
| Standard deviations | | | | | | |  |
| Diagnostic cost | | 0.016** (0.001) | | 0.012** (0.001) | 0.015** (0.001) | |  |
| OSA follow up frequency | | 0.221** (0.054) | | -0.011 (0.100) | 0.144* (0.071) | |  |
| OSA initial assessment provider | Self-assessed | 0.744** (0.174) | | 0.444 (0.255) | -0.725** (0.134) | |  |
|  | GP | -0.573* (0.225) | | -0.443 (0.279) | 0.560** (0.180) | |  |
|  | Pharmacist or CPAP shop rep | -0.798** (0.183) | | 0.823** (0.245) | 0.802** (0.155) | |  |
|  | No one | 4.753** (0.248) | | 3.560** (0.281) | 4.325** (0.181) | |  |
| Sleep study setting | Private Hospital | -0.196 (0.335) | | 0.465 (0.277) | -0.131 (0.244) | |  |
|  | GP | -0.112 (0.319) | | 0.555* (0.219) | 0.541** (0.157) | |  |
|  | Public Hospital | 0.867** (0.155) | | 0.733** (0.183) | 0.767** (0.122) | |  |
|  | Pharmacy or CPAP shop | -0.242 (0.341) | | -0.527* (0.217) | 0.003 (0.236) | |  |
| Wait time | No wait (same day) | -0.099 (0.178) | | 0.211 (0.255) | 0.091 (0.160) | |  |
|  | Within 1 week | 0.335 (0.204) | | -0.237 (0.254) | -0.242 (0.172) | |  |
|  | 1 week to 1 month | 1.131** (0.311) | | -0.699 (0.409) | 0.996** (0.251) | |  |
|  | 1 month to 3 month | -0.340 (0.411) | | 0.923** (0.258) | 0.769** (0.201) | |  |
| Result interpretation and treatment recommendation provider | Sleep physician | -0.061 (0.181) | | -0.055 (0.311) | 0.081 (0.145) | |  |
|  | GP | -0.300 (0.336) | | 0.411 (0.312) | 0.301 (0.220) | |  |
|  | Pharmacist or CPAP shop rep | 0.582* (0.230) | | 0.315 (0.243) | 0.413** (0.160) | |  |
|  | No one | 1.238** (0.158) | | -0.326 (0.409) | 1.124** (0.137) | |  |
| Treatment option | CPAP therapy | 0.655* (0.277) | | 0.523* (0.231) | 0.660** (0.186) | |  |
|  | Mouthguard | 0.727** (0.260) | | -0.593 (0.324) | 0.923** (0.172) | |  |
|  | Lifestyle changes | 0.667** (0.196) | | 0.035 (0.443) | -0.585** (0.164) | |  |
|  | No treatment | 0.544** (0.161) | | 0.706** (0.190) | -0.487** (0.171) | |  |
| OSA ongoing care provider | Sleep physician | 0.583* (0.238) | | 0.045 (0.278) | 0.273 (0.201) | |  |
|  | GP | 0.400 (0.307) | | 0.605** (0.200) | -0.403* (0.179) | |  |
|  | Pharmacist or CPAP shop rep | 0.575** (0.181) | | 0.751** (0.254) | -0.632** (0.178) | |  |
|  | Dentist or ENT surgeon | 0.820** (0.155) | | -0.325 (0.332) | -0.647** (0.144) | |  |
| LL^b^ | | | -5389.6 | -2759.93 | | -8243.9 | |
| AIC^c^ | | | 10883.2 | 5623.861 | | 16591.8 | |
| BIC^d^ | | | 11141.14 | 5838.685 | | 16868.58 | |
| N | | | 1054 | 460 | | 1514 | |
| Obs | | | 22,134 | 9,660 | | 31,794 | |
| LL for S-L test^e^ | | |  | | | 188.740 | |

*CPAP=* Continuous positive airway pressure; *ENT=* Ear, Nose and Throat; *GP=* General practice; *OSA*= Obstructive sleep apnoea; *SD*= standard deviation

Note: Figures are coefficients (standard errors) */** Coefficient statistically significant at 5%/ 1% level of significance

^a^ In the simulation-based technique, 500 Halton draws were run

^b^ LL = Log-likelihood ^c^ AIC = The Akaike information criterion ^d^ BIC = Bayesian information criterion

^e^ S&L test = Swait-Louviere Test is calculated using the sum of LL statistics of OSA diagnosed and high-risk groups subtracted from the LL of the pooled sample (-8243.9). The X^2^ statistics from the S&L likelihood ratio tests for equality of model parameters for both groups (188.740) were higher than the X^2^ critical value of 40.113 (based on 5% level of significance and 27 degrees of freedom). Hence, the data relating to both groups were analysed separately as analysing by the pooled sample is inappropriate.

**Table S7** Uncorrelated Mixed Logit regression estimates (nrep=500, n=1,454) ^a^

| Attribute and levels | | | Undiagnosed, high-risk OSA | Diagnosed with OSA | | Total sample | |
| --- | --- | --- | --- | --- | --- | --- | --- |
| OSA initial assessment provider | Self-assessed | | 0.511 (0.109)** | 0.583 (0.141)** | | 0.514 (0.086)** | |
|  | GP | | 1.409 (0.133)** | 0.853 (0.183)** | | 1.179 (0.108)** | |
|  | Pharmacy or CPAP shop | | 0.549 (0.138)** | 0.414 (0.188)* | | 0.539 (0.109)** | |
|  | No one | | 1.455 (0.424)** | -0.407 (0.597) | | 0.649 (0.334) | |
| Sleep study setting | Private Hospital | | 1.361 (0.408)** | 1.499 (0.581)** | | 1.230 (0.321)** | |
|  | GP | | 1.532 (0.336)** | 1.274 (0.476)** | | 1.273 (0.263)** | |
|  | Public Hospital | | 1.185 (0.342)** | 1.600 (0.493)** | | 1.172 (0.270)** | |
|  | Pharmacy or CPAP shop | | 1.200 (0.373)** | 1.353 (0.529)* | | 1.116 (0.293)** | |
| Diagnostic cost | (continuous) | | -0.017 (0.001) ** | -0.012 (0.001)** | | -0.015** (0.001) | |
| Wait time | No wait (same day) | | 0.958 (0.137)** | 0.612 (0.175)** | | 0.787 (0.102)** | |
|  | Within 1 week | | 0.787 (0.141)** | 0.409 (0.181)* | | 0.632 (0.108)** | |
|  | 1 week to 1 month | | 0.477 (0.186)* | 0.526 (0.235)* | | 0.479 (0.135)** | |
| Result interpretation and treatment recommendation provider | Sleep physician | | 0.927 (0.160)** | 0.881 (0.214)** | | 0.880 (0.124)** | |
|  | GP | | 0.549 (0.144)** | 0.358 (0.190) | | 0.506 (0.110)** | |
|  | Pharmacist or CPAP shop rep | | 0.448 (0.133)** | 0.620 (0.178)** | | 0.507 (0.104)** | |
|  | No one | | 0.419 (0.171)* | 0.477 (0.221)* | | 0.413 (0.130)** | |
| Treatment option | CPAP therapy | | 0.725 (0.147)** | 0.981 (0.202)** | | 0.765 (0.115)** | |
|  | Mouthguard | | 0.896 (0.158)** | 0.796 (0.215)** | | 0.823 (0.124)** | |
|  | Lifestyle changes | | 0.861 (0.151)** | 0.839 (0.205)** | | 0.826 (0.116)** | |
|  | No treatment | | 0.458 (0.134)** | 0.352 (0.180) | | 0.373 (0.104)** | |
| OSA follow up frequency | (continuous) | | -0.133 (0.039)** | -0.068 (0.051) | | -0.079 (0.029)** | |
| OSA ongoing care provider | Sleep physician | | 1.217 (0.217)** | 1.131 (0.286)** | | 1.054 (0.163)** | |
|  | GP | | 1.233 (0.248)** | 0.791 (0.332)* | | 0.923 (0.186)** | |
|  | Pharmacist or CPAP shop rep | | 0.820 (0.227)** | 0.471 (0.314) | | 0.602 (0.173)** | |
|  | Dentist or ENT surgeon | | 0.500 (0.247)* | 0.463 (0.336) | | 0.422 (0.189)* | |
| Standard deviations | | | | | | |  |
| Diagnostic cost | | 0.016 (0.001)** | | 0.013 (0.002)** | 0.015 (0.001)** | |  |
| OSA follow up frequency | | -0.305 (0.049)** | | -0.107 (0.096) | 0.148 (0.069)* | |  |
| OSA initial assessment provider | Self-assessed | 0.626 (0.184)** | | 0.464 (0.289) | 0.550 (0.141)** | |  |
|  | GP | 0.358 (0.304) | | 0.543 (0.294) | 0.503 (0.215)* | |  |
|  | Pharmacist or CPAP shop rep | 0.664 (0.205)** | | 0.861 (0.267)** | -0.701 (0.154)** | |  |
|  | No one | 4.660 (0.230)** | | 3.820 (0.307)** | 4.467 (0.195)** | |  |
| Sleep study setting | GP | -0.346 (0.266) | | -0.503 (0.281) | 0.527 (0.161)** | |  |
|  | Public Hospital | 0.677 (0.214)** | | 0.496 (0.291) | 0.651 (0.143)** | |  |
|  | Pharmacy or CPAP shop | -0.143 (0.269) | | 0.515 (0.294) | -0.376 (0.160)* | |  |
| Wait time | 1 week to 1 month | 1.166 (0.316)** | | -0.748 (0.586) | 0.856 (0.283)** | |  |
|  | 1 month to 3 months | 0.707 (0.327)* | | 1.091 (0.304)** | 0.700 (0.246)** | |  |
| Result interpretation and treatment recommendation provider | GP | 0.518 (0.238)* | | -0.175 (0.425) | -0.060 (0.303) | |  |
|  | No one | 1.258 (0.164)** | | 0.377 (0.479) | 1.042 (0.173)** | |  |
| Treatment option | CPAP therapy | -0.546 (0.320) | | -0.513 (0.376) | 0.826 (0.199)** | |  |
|  | Mouthguard | 0.690 (0.259)** | | 1.108 (0.276)** | 0.852 (0.190)** | |  |
|  | Lifestyle changes | 0.899 (0.146)** | | 0.134 (0.384) | 0.439 (0.200)* | |  |
|  | No treatment | 0.660 (0.163)** | | 0.741 (0.260)** | 0.441 (0.204)* | |  |
| OSA ongoing care provider | Sleep physician | 0.535 (0.244)* | | 0.370 (0.240) | 0.393 (0.186)* | |  |
|  | Pharmacist or CPAP shop rep | -0.623 (0.207)** | | 0.971 (0.254)** | 0.706 (0.151)** | |  |
|  | Dentist or ENT surgeon | 0.916 (0.150)** | | -0.469 (0.257) | 0.545 (0.166)** | |  |
| LL^b^ | | | -5244.43 | -2444.78 | | -7778.32 | |
| AIC^c^ | | | 10592.85 | 4993.555 | | 15660.64 | |
| BIC^d^ | | | 10849.75 | 5203.772 | | 15935.31 | |
| N | | | 1033 | 421 | | 1454 | |
| Obs | | | 21,693 | 8,841 | | 30,534 | |
| LL for S-L test^e^ | | |  | | | 178.234 | |

*CPAP=* Continuous positive airway pressure; *ENT=* Ear, Nose and Throat; *GP=* General practice; *OSA*= Obstructive sleep apnoea; *SD*= standard deviation

Note: Figures are coefficients (standard errors) */** Coefficient statistically significant at 5%/ 1% level of significance

^a^ In the simulation-based technique, 500 Halton draws were run

^b^ LL = Log-likelihood ^c^ AIC = The Akaike information criterion ^d^ BIC = Bayesian information criterion

^e^ S&L test = Swait-Louviere Test is calculated using the sum of LL statistics of OSA diagnosed and high-risk groups subtracted from the LL of the pooled sample (7778.32). The X^2^ statistics from the S&L likelihood ratio tests for equality of model parameters for both groups (178.234) were higher than the X^2^ critical value of 40.113 (based on 5% level of significance and 27 degrees of freedom). Hence, the data relating to both groups were analysed separately as analysing by the pooled sample is inappropriate

## References

1. *Ngene 1.1.1 User Manual & Reference Guide* [computer program]. Australia 2012.

2. Carlsson F, Martinsson P. Design techniques for stated preference methods in health economics. *Health economics.* 2003;12(4):281-294.

3. Krucien N, Gafni A, Fleury B, Pelletier-Fleury N. Patients’ with obstructive sleep apnoea syndrome (OSAS) preferences and demand for treatment: a discrete choice experiment. *Thorax.* 2013;68(5):487-488.

4. Krucien N, Le Vaillant M, Pelletier-Fleury N. What are the patients' preferences for the Chronic Care Model? An application to the obstructive sleep apnoea syndrome. *Health Expectations.* 2015;18(6):2536-2548.

5. Kaambwa B, Lancsar E, McCaffrey N, et al. Investigating consumers' and informal carers' views and preferences for consumer directed care: A discrete choice experiment. *Social Science & Medicine.* 2015;140:81-94.

6. Kaambwa B, Ratcliffe J, Shulver W, et al. Investigating the preferences of older people for telehealth as a new model of health care service delivery: A discrete choice experiment. *Journal of telemedicine and telecare.* 2017;23(2):301-313.

7. Appleton SL, Gill TK, Lang CJ, et al. Prevalence and comorbidity of sleep conditions in Australian adults: 2016 Sleep Health Foundation national survey. *Sleep health.* 2018;4(1):13-19.

8. Hole AR. Small-sample properties of tests for heteroscedasticity in the conditional logit model. *Economics Bulletin.* 2006;3(1).

9. McFadden D, Train K. Mixed MNL models for discrete response. *Journal of Applied Econometrics.* 2000;15(5):447-470.

10. Hensher DA, Rose JM, Greene WH. *Applied choice analysis: a primer.* Cambridge University Press; 2005.

11. Hole AR. Fitting mixed logit models by using maximum simulated likelihood. *The Stata Journal.* 2007;7(3):388-401.

12. Bhat CR. Simulation estimation of mixed discrete choice models using randomized and scrambled Halton sequences. *Transportation Research Part B: Methodological.* 2003;37(9):837-855.

13. Czajkowski M, Budziński W. Simulation error in maximum likelihood estimation of discrete choice models. *Journal of choice modelling.* 2019;31:73-85.

14. Lancsar E, Louviere J. Conducting discrete choice experiments to inform healthcare decision making. *Pharmacoeconomics.* 2008;26(8):661-677.

15. Hensher DA. Accounting for scale heterogeneity within and between pooled data sources. *Transportation Research Part A: Policy and Practice.* 2012;46(3):480-486.

16. Chai-Coetzer CL, Antic NA, Rowland LS, et al. A simplified model of screening questionnaire and home monitoring for obstructive sleep apnoea in primary care. *Thorax.* 2011;66(3):213-219.

17. McCaffrey N, Kaambwa B, Currow DC, Ratcliffe J. Health-related quality of life measured using the EQ-5D–5L: South Australian population norms. *Health and Quality of Life Outcomes.* 2016;14(1):133.

18. Finn L, Young T, Palta M, Fryback DG. Sleep-disordered breathing and self-reported general health status in the Wisconsin Sleep Cohort Study. *Sleep.* 1998;21(7):701-706.

19. Baldwin CM, Griffith KA, Nieto FJ, O'Connor GT, Walsleben JA, Redline S. The Association of Sleep-Disordered Breathing and Sleep Symptoms with Quality of Life in the Sleep Heart Health Study. *Sleep.* 2001;24(1):96-105.
